# Development of Adult Worms and Granulomatous Pathology Are Collectively Regulated by T- and B-Cells in Mice Infected with *Schistosoma japonicum*

**DOI:** 10.1371/journal.pone.0054432

**Published:** 2013-01-22

**Authors:** Hongbin Tang, Zhenping Ming, Rong Liu, Tao Xiong, Christoph G. Grevelding, Huifeng Dong, Mingsen Jiang

**Affiliations:** 1 Laboratory Animal Center, medicine school, Wuhan University, Wuhan, China; 2 Department of Medical Parasitology, School of Basic Medical Science, Wuhan University, Wuhan, China; 3 Institute for Parasitology, Justus-Liebig-University, Giessen, Germany; Temple University School of Medicine, United States of America

## Abstract

*Schistosoma* blood flukes, which cause schistosomiasis affecting 200 million people in the world, are dependent on signals from host CD4^+^ T cells to facilitate parasite growth and development in the mammalian host and to induce Th2-biased inflammatory granulomas. B cells, however, are reported to down-regulate granulomatous pathology in schistosomiasis, but not to affect the development of blood flukes together with CD4^+^ T lymphocytes. Thus it is not clear whether B cells mediate parasite development, reproduction and egg granuloma formation of schistosomes without the help of CD4^+^ T lymphocytes. Using mice that have severe combined immunodeficiency (scid) and mice lacking T cells (nude), we found that the absence of B cells can more seriously hamper the development and paring of adult worms, but granuloma formation of *Schistosoma japonicum* in scid mice was not down-regulated comparing with that in nude mice. The level of IL-10 in the sera of nude mice was significantly higher than of scid mice at 43 days post infection (p.i.). Thus multiple mechanisms of immune modulation seem to be involved in parasite development and reproduction by helminth-induced regulatory B cells. Our findings have significance for understanding the molecular connections between schistosomes and T- and B-cells, indicating that more research is needed to develop efficient vaccine-based therapies for schistosomiasis.

## Introduction

Schistosomes are metazoan parasites which infect more than 200 million people worldwide, and about 700 million are at risk of infection [1]. The host-parasite relationship (between schistosomes and mammal hosts) is complex and long-lived. As the only members of the trematodes, schistosomes have evolved separate sex. Furthermore, mating is required to induce the sexual maturation of the female as a prerequisite for egg production. The latter finally leads to inflammatory processes in the gut and the liver culminating in the formation of granulomas. The traditional opinion is that parasites may damage their hosts, and the host resists against the invading parasites by its immune response. However, some studies have suggested that host factors including endocrine and immune signals can modulate parasite development and maturation [2], [3], [4], [5].

It was recently shown that schistosome co-opt CD4^+^ T cell–driven mechanisms to facilitate parasite development and egg excretion [4], [6], [7]. The role of CD4^+^ T cells in this process may be limited to provide non-cognate help for mononuclear phagocyte function [8]. However, schistosome development was normal in immunoglobulin heavy chain (Igh)6^−/−^ mice only lacking B cells [9], [10]. Egg granuloma formation was dependent on sensitized CD4^+^ T lymphocytes, with both Th1 and Th2 subsets participating in different stages during the development of the lesions [11], [12], [13].

In this study we investigated whether the CD4^+^ T lymphocyte has influence on development of *S.japonicum*, and whether the B lymphocyte population was responsible for modulating schistosome development and granulomatous pathology in the absence of T cells. To address this question we conducted a detailed analysis of the development of worms in the different stages post infection, characterizing besides growth and reproduction also granuloma formation in a immunodeficiency mouse model for *Schistosoma japonicum* infection. Parasite development was evaluated by analyzing three separate parasitological parameters: worm length; the proportion of female parasites participating in pairs; and the number of eggs deposited in the liver by each parasite pair. Worm length was used as a measure of parasite growth during the pre-patent period, whereas pairing and egg production were used to assess parasite sexual maturation and subsequent reproductive activity, respectively. To assure the absence of B or T cells, we determined the amount of specific B- and T-lymphocyte populations in peripheral blood by flow cytometry (FCM), and the presence of IL-10 in peripheral blood by ELISA. Interestingly, our data indicate for the first time that schistosomes not only take advantage from T cell immune signals, but also from B-cells to manage development.

## Materials and Methods

### Parasite Maintenance and Infection

Six to eight weeks old male BALB/c mice, nude mice, and scid mice in BALB/c background, originally purchased from laboratory animal center of Wuhan university, were maintained by Wuhan university center for animal experiments(WUCAE). All animals were housed under specific pathogen-free conditions in an American Association for the Accreditation of Laboratory Animal Care internationally approved facility. A minimum of 8 mice were used in each experimental group, unless otherwise indicated. Cercariae of *S. japonicum* (strain from Jiangsu Province, China) were shed from *Oncomelania hupensis* snails [14]. Miracidia that obtained from Eggs of livers in rabbits at 45 days post infection (p.i.) infect snails after incubated for 2–4 hours, and the snails were incubated for 75 days at 25°C. Mice were infected percutaneously via the abdomen skin with 30±1 cercariae. Each strain of mice was divided into three groups. Mice were killed at days 29, 36, or 43 p.i., respectively.

### Parasite Analysis

Parasites were perfused in hepatic portal veins [15] and immediately fixed in 4% neutral buffered formaldehyde to prevent pairs from dissociating. The collected worms were counted under a dissecting microscope. All fixed worms were photographed using a LY-WN-HPCCD,5M Pixels High-Speed HPCCD-5 Color Microscope Camera connected to a Vistavision trinocular dissecting microscope at 10× magnification. Parasite length was measured in digital images using “Image-Pro Plus 5.0” software (MediaCybernetics).

### Determination of Egg Production

Following perfusion, livers were collected and weighed, and the large left lobe of each liver was digested at 37°C for 3 h in 20 ml/mg of 5% potassium hydroxide (KOH). Three replicates of 0.1 ml samples were counted from each digest and the mean value determined. Egg production per schistosome pair was calculated by dividing the total number of eggs from each mouse liver by the number of parasite pairs recovered [8], [16].

### Histologic Evaluation of Granuloma Formation

The remaining right liver lobe of each mouse was fixed for histological examination. Livers were fixed in 10% buffered formaldehyde, embedded in paraffin and cut into 5-µm sections. The sections were stained with hematoxylin and eosin (H&E). Representative H&E-stained liver sections from each animal were scanned under ×400 magnifications with a compound microscope. For every granuloma containing a single egg, the granuloma size was determined by two perpendicular diameters at the mid-transmiracidial level of the egg [17]. Digital images were analyzed using “Image-Pro Plus 5.0” software.

### Flow Cytometry

For analysis of T cell activation in cells recovered from peripheral blood, expression of CD4 was examined by flow cytometry, after gating on CD3^+^ cells. Cells isolated from peripheral blood were surface-labeled with FITC-conjugated antibodies to CD3, and PE-conjugated antibodies to CD4 (BD Biosciences). For analysis of B- cell populations, cells were stained with FITC-conjugated anti-CD45R and PE-conjugated anti-CD45. Cells expressing CD45R were examined by flow cytometry, after gating on CD45 cells. All samples were analyzed by using a LSR II Optical Bench flow cytometer with FACSDiva (BD Biosciences) and CellQuest software (BD Biosciences).

### Analysis of Cytokine Production

Cytokines were measured by ELISA using Immulon 2HB plates (Thermo) according to the manufacturerś guidelines. Capture and detection antibodies for IL-10 were purchased from R&D. Serum IL-10 was measured using an *in vivo* cytokine capture assay (IVCCA) as previously described [18]. ELISA plates were washed with 0.05% Tween 20 in PBS (PBST) and blocked with 5% non-fat milk in PBST. Recombinant cytokine standards (R&D) were used to assess the quantities of cytokines in supernatants using a standard curve, with OD acquired at 450 nm in an ELISA reader.

### Statistical Analysis

ANOVA was applied to determine the statistical significance of differences in median values from different experimental groups. In experiments involving 3 different experimental groups, LSD and Tamhane’s multiple comparison tests were employed to evaluate differences between each pair of experimental groups. Statistical analyses were performed with PASW Statistics Version 18.0 software (SPSS Inc.). P-values of less than 0.05 were considered statistically significant. Experiments were repeated twice, with 8 animals per group.

### Ethics Staement

All animal studies were performed in accordance with protocols approved by the WUCAE Institutional Animal Care and Use Committee.

## Results

### CD4^+^ Lymphocytes in Peripheral Blood Facilitate the Growth of *S. japonicum*

**Figure 1:**
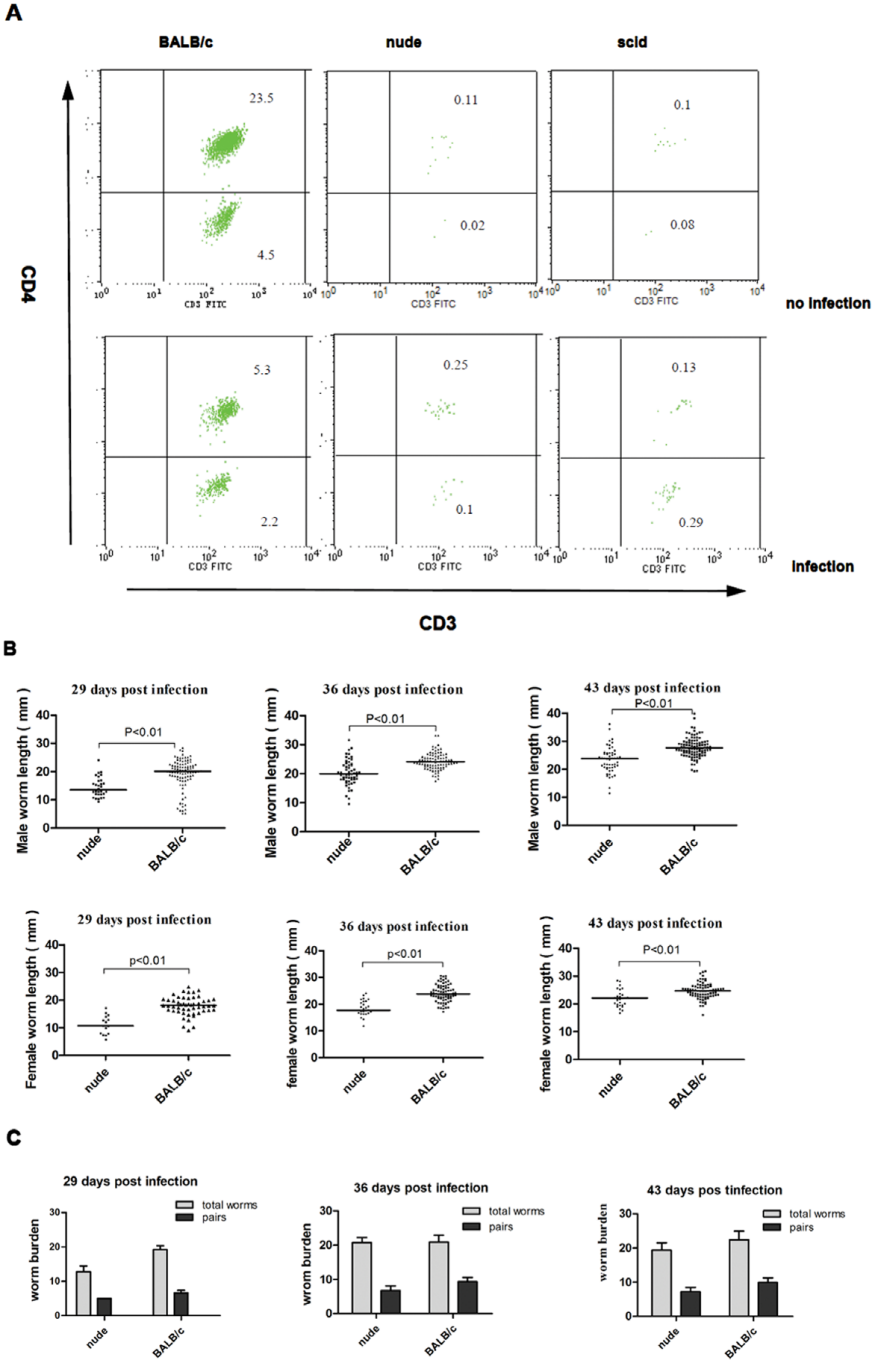
The effects of CD4^+^ T cell deficiency on the development of worms. (A) Flow cytometric analysis of peripheral blood CD4^+^ lymphocytes in scid, nude and BALB/c mice, recovered at 43 days p.i. Presented are % of cells detected in CD3 gate as a mean from one of 2 experiments with 4 mice per group. (B) Parasites were recovered from the portal tract at 29, 36, 43 days p.i., and male and female worm sizes were determined based on digital micrographs. Mean values are represented by horizontal bars. (C) Total number of worms and worm pairs in nude and BALB/c mice at 29, 36, 43 days p.i… Data are represented as median± SEM.

**Figure 2:**
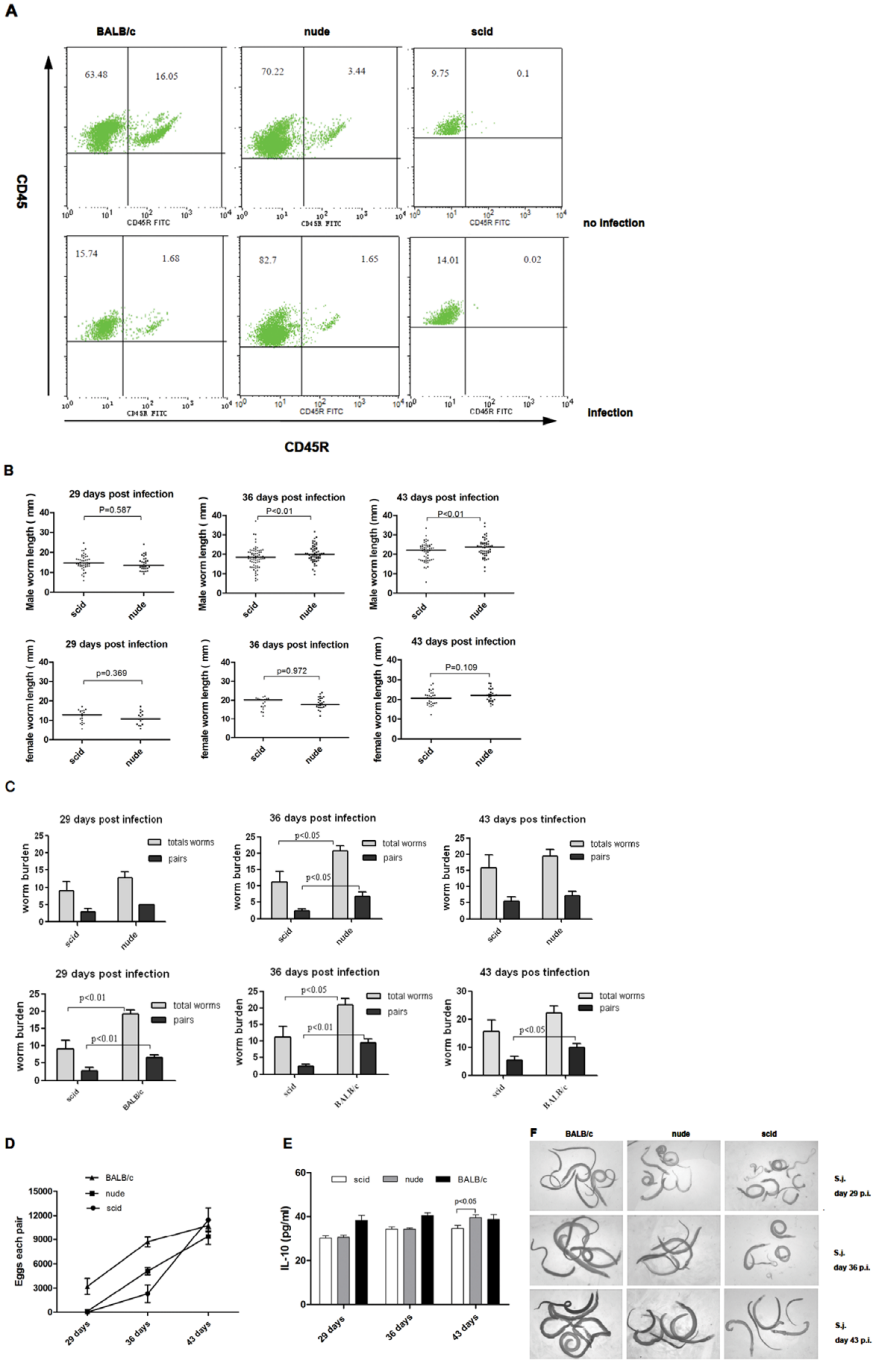
B cells and T cells play crucial roles for development and reproduction of schistosomes. (A) Flow cytometric analysis of peripheral blood B lymphocytes in scid, nude and BALB/c mice; peripheral blood was recovered at 43 days p.i… Presented are, % of cells in the CD45R gate as mean from one of two experiments), with 4 mice per group. (B) Sizes of parasites from scid and nude mice were measured from digital micrographs. (C) Total number of worms and worm pairs recovered from scid, nude, or BALB/c mice were compared at 29, 36, 43 days p.i… Data are represented as median± SEM. (D) Egg production by schistosome pairs, as in C. (E) IL-10 cytokine secretions, measured by ELISA, in scid (white bars), nude (gray bar) and BALB/c (black bar) mice at 29, 36, 43 days p.i. (F) Representative micrographs of parasites recovered from BALB/c mice, nude mice, or scid mice at the time points indicated p.i. (10× magnification).

To evaluate the role of CD4^+^ T cells in schistosome growth and reproduction, we examined the body length of sexual worms (Figure 1B) and the population of lymphocytes in nude mice that lack a thymus and in BALB/c mice as the control (Figure 1A). Analysis of peripheral lymphocyte populations by FCM revealed the lack of CD4^+^ T cells in nude mice compared with immunocompetent BALB/c mice. In nude mice, schistosome development was impaired relative to wild type mice that have normal CD4^+^ T cells, as determined by measuring the parasite sizes (Figure 1B). Furthermore, the observed differences of worm length between the above two groups were neither influenced by the time point of recovery nor by gender. The overall number of worms recovered from nude mice were less than the control group (Figure 1C), especially female worms (Figure 1B). So the pairing rates in total worms from mice were not very high and the differences were no significant between the above two groups (Figure 1C). However, with respect to the number of eggs per couple in the liver of nude mice and BALB/c mice at 29, 36, 43 p.i. (Figure 2D), none of the differences was significant. Taken together, the data suggested that CD4^+^ T cells have no influence on reproduction, but on the growth of *S. japonicum*.

### B cells Promote Schistosome Growth in the Absence of CD4^+^ T cells

Since non-cognate CD4^+^ T cells were shown to facilitate parasite development [8], and since CD4^+^ T cell-mediated granulomatous pathology in schistosomasis is downregulated by a B cell-dependent mechanism [10], we hypothesized that B cells may also play a role in promoting schistosome development and reproduction. To test whether B cells have similar functions as CD4^+^ T cells, we examined *S. japonicum* development also in scid mice that have normal macrophages, but lack of functional B and T lymphocytes [19], [20]. Indeed, the influence on development was observed. However, the extent to which parasite growth was limited varied between the different strains. the suppression of parasite growth was most serious in scid mice, and then in nude mice. As expected, the mean length of male parasites from scid mice was significantly shorter than from nude mice at different time points (36, 43 days p.i.), but there was no significant differences of the body length of female parasites (Figure 2B). We have observed that the total number of worms and the number of worm pairs recovered from scid mice were lower than those obtained from BALB/c or nude mice perfused at different time points (29, 36, 43 days) p.i.(Figure 2C). When compared with nude mice at 36 days p.i., the infected scid mice showed significantly decreased egg numbers per pair (Figure 2D), a parameter indicative for the reproductive capacity of the parasite. Scid and nude mice were quantitatively indistinguishable in terms of tissue egg recovery at 43 days p.i., which is consistent with previous studies [21]. Nevertheless, the decline of worm pairing led to decrease in the total number of eggs in liver, indicating that B cells exert an influence on the growth of *S. japonicum* during primary infection.

Because schistosome infection protected mice from anaphylaxis via IL-10-producing B cells [22], and since IL-10 blocked the development of resistance to re-infection with schistosome [23], the levels of the regulatory cytokine IL-10 were elevated in infected mice (Figure 2E). A significant decrease of worm-specific IL-10 production in scid and nude mice serum levels was determined at two time points (29 and 36 days p.i.). However, a significant decrease IL-10 serum levels was only observed in scid mice 43 days p.i., whereas no differences of IL-10 levels were found in nude mice and BALB/c mice at 43 days p.i. These data suggest that depletion of B cells lead to reduce IL-10 levels in peripheral blood, even if the hosts were stimulated by helminth.

### B cells are not Indispensable in the Egg Granuloma Formation in SCID Mice

**Figure 3:**
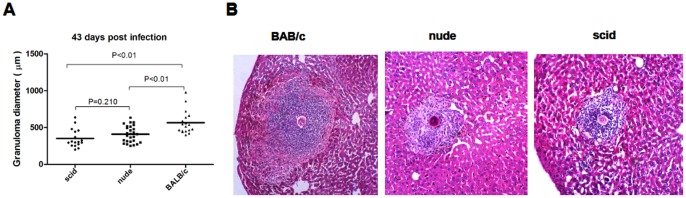
Hepatic granuloma size and fibrosis is reduced in nude mice versus BALB/c mice, but the same as scid mice. (A) granuloma sizes calculated from isolated granulomas at 43 days after infection calculated from three separate granulomas per individual animal. (B) a typical well-formed and multi-cellular granuloma with minimal hepatocyte necrosis (×200) includes a large number of eosinophils in BALB/c. granuloma with a much reduced cellular infiltration and obvious hepatocyte necrosis surrounding egg show the relative selective depletion of eosinophils and cellularity in nude and scid, but there is no difference significant between the two.

To test whether host B cells contribute to granuloma formation without Th1 and Th2 subsets participating, we examined *S. japonicum*-derived hepatic granuloma formation by comparatively measuring of granuloma diameters in infected scid mice, nude mice, or BALB/c mice. No typical granuloma formation was observed for any group at days 29 and 36 p.i., time points where adult worms have reached the liver and start pairing as a prerequisite for subsequent egg production. Histological examinations at day 43 p.i. showed egg granulomas, which significantly differed between the different mouse strains used. Whereas BALB/c mice exhibited multi-cellular granulomas with a large number of eosinophils and minimal hepatocyte necrosis, granulomas from nude mice as well as scid mice were of smaller size showing reduced cellular infiltrations but obvious hepatocyte necrosis in the flanking liver tissue that was not observed in BALB/c mice (Figure 3A). The nude mice and scid mice developed the diameter of the granulomas that were smaller than those in the BALB/c group by 27% and 38%, however, the granulomas diameter between nude and scid group was insignificant (P>0.05).

Within the granulomas a significant diminution in the number of eosinophils was noted in nude compared with BALB/c mice. Lymphocytes and other mononuclear cells also appeared to be decreased in these granulomas, although to a lesser degree. In addition, hepatocyte necrosis in the vicinity of eggs was obvious in nude and scid, but minimal in BALB/c mice (Figure 3B).

## Discussion

In this study three mouse strains with different immunogenetic background were infected with *S. japonicum* to study effects on schistosome development and granuloma formation in the host. We have demonstrated that T cell-deficient mice as well as animals lacking functional T and B cells negatively influenced the growth of the parasite. Furthermore, we discovered a more serious impairment of schistosome development in animals lacking functional T and B cells than in mice only deficient in T cells. Pervious studies showed that schistosomes require host CD4^+^ T cells for normal development [6], [24], [25], which also mediate egg deposition via the gut wall [18], [26]. Development and sexual maturation of schistosomes were also shown to be influenced by components of the adaptive immune system [25]. Our results from *S. japonicum* infection in immune-deficient hosts are in agreement with previous studies suggesting that the growth of this parasite is influenced by immune signals. However, Unlike pervious researches [7], [17], we have observed that parasite development significantly differed between nude and BALB/c mice at 36 and 43 days p.i. The most likely explanation lies in a source of hosts and *S. Japonicum* that different sources host’s thymus glands have different immunological reactions to infection of pathogens, and development degree of different origins cercaria is different in same host. Our data indicate that CD4^+^ T cells are the critical immune element for normal *S.japonicum* development, and the absence of CD4^+^ T cells impairs the growth of this parasite in mice.

It was showed that CD4^+^ T cells had no effect on reproduction of worms. parameters of pairing and egg production of worms were no obvious differences in nude and BALB/c mice, even if eggs each pair were lower in nude mice than in normal control group at 29 and 36 days p.i. It indicated that CD4^+^ T cells only involved in the regulation of parasite growth. some researches showed that adoptive transfer of activated T-cells into scid mice were not sufficient to restore schistosomes eggs per pair to normal levels [3] and no defect in parasite paring were reported [27], which indicated that activated T cells did not directly influence the reproduction of blood fluke. We conclude that CD4^+^ T cells will only facilitate schistosome growth but not to affect schistosome reproduction including paring and egg production.

Someone thought that B cells, as the sentinels of the adaptive immune system, are not responsible for modulating schistosome development [6]. Nevertheless, in our investigation, schistosome development, such as male worms growth, had more inhibition in sicd mice than nude mice, revealing that B cells are important for parasite development without CD4^+^ T lymphocytes in the host. Our data demonstrate that the absence of T or B cells not only impairs schistosome growth, but also inhibits parasite paring. Together with previous finding that adoptive transfer of wild-type CD4^+^ T cells into RAG-1^−/−^ animals was sufficient to restore *S. japonicum* development to normal levels [25] and schistosomes do not respond directly to CD4^+^ T cells, as their absence can be bypassed completely by direct stimulation of innate immune responses [8]., our study implicate that B cells possibly are a by-pass or backup of CD4^+^ T lymphocyte regulation in the worm development. In normal circumstance, schistosome development have been played a major role by CD4^+^ T lymphocytes. In the absence of T cells, B cells are activated by helminth infection and produce cytokines to regulate helminth development. In support of this, IL-10-producing Bregs can also be activated by T-cell-independent stimuli, particularly TLR ligands [28]. Thus, it is tempting to speculate that helminths may be able to induce the activity of regulatory B cells [29]. Furthermore, in response to many stimuli, including CD40 ligation, Be-1, Be-2 cells and human peripheral blood B cells produce IL-10 make IL-10 [30], [31]. For example, *S. mansoni*-induced regulatory B cells can attenuate allergic disease through an IL-10-dependent mechanism [22], [32], [33]. *S. mansoni* infection promotes the expansion of peritoneal B1 cells and splenic B cells, and egg-derived oligosaccharides promote B cell proliferation and IL-10 production [34], [35]. In this study we also measure the level of IL-10 in the peripheral blood of infected mice. Our findings correspond to results of previous studies where IL-10-competent B cells were the prevalent source of IL-10, but not other cytokines [36], suggesting that schistosome infections of mice stimulate B cells to produce IL-10, which may be involved in the regulation of parasite growth and paring.

Our experiment showed that parasite paring was inhibited in scid mice, but not in nude mice. In addition, parasite development was normal in Igh-6^−/−^ mice lacking B cells [6]. It showed that schistosome pairing was double modulated by both T and B cells, and one deficiency of them had no impact on schistosome pairing. They had also suggested B cells are a bypass or backup of T cells in the regulation of schistosome development. We have also observed that total number of eggs reduced in the livers of scid mice relative to nude and BALB/c mice at 29 and 36 days p.i., but there is no significant difference in eggs each pair among them. This has been interpreted to mean that the absence of B and T cells decreasing the rate of parasite pairing leads to drop total number of eggs in the livers, but not influence eggs each pair. In other words, schistosome reproduction is not significantly affected by B and T cells in the host.

The process of egg granuloma formation is dependent on sensitized CD4^+^ T lymphocytes. A variety of different mechanisms include cross-regulation by cytokines produced by Th1 or Th2 cells [36], [37], [38], [39], and the development of antiidiotypic Ab or T cells [40], [41]. When quantitatively infected with *S.japonicum,* scid mice as well as nude mice displayed down-regulation of the granuloma formation typically observed in BALB/c mice. Surprisingly, this augmented pathology occurring in scid mice is different from in B cell-deficient mice or in mice by blockade of the receptor for IL-10 during chronic schistosomiasis [42] where granuloma volumes are significantly larger than that of the WT mice [10]. And blockage of IL-10 in the in vitro granuloma assay lead to a significant increase in granuloma size with cells from intestinal patients but not with individuals in the acute phase or with the hepatosplenic form of schistosomiasis [43]. However, as previously reported [7], [44], [45], T cell-deprived mice infected with *S.japonicum* develop greatly reduced egg granulomas and disseminated hepatocelluar lesions in the liver compared with that in intact control mice. Thus, these findings indicate that B cells may mediate CD4^+^ T cell to down-regulate the granuloma formation during chronic schistosomiasis, and this pathologic consequence is not directly influenced by B cells and IL-10 in the acute phase.

In conclusion, this study demonstrates that B cells and CD4^+^ T lymphocytes cooperatively facilitate development of blood flukes in mice. Furthermore, schistosome development is hampered more seriously in the mice lacking T and B cells mice than in the mice lacking T cells alone. As a regulatory mechanism with a bypass or backup, B cells and T cells double improve schistosome development. However, B cells contributing to the regulation of granulomatous pathology caused by schistosome infection seem to depend on CD4^+^ T lymphocytes. Thus, multiple mechanisms of immune modulation involving in parasite development by helminth-induced regulatory B cells are likely to exist.
