# Correction: Development of Adult Worms and Granulomatous Pathology Are Collectively Regulated by T- and B-Cells in Mice Infected with *Schistosoma japonicum*

**DOI:** 10.1371/annotation/40c28bf3-6dae-4d1e-b74c-a6a8f2450a8a

**Published:** 2013-06-07

**Authors:** Hongbin Tang, Zhenping Ming, Rong Liu, Tao Xiong, Christoph G. Grevelding, Huifeng Dong, Mingsen Jiang

One of the affiliations for the first author was not indicated. Hongbin Tang is affiliated with: 1 Laboratory Animal Center, medicine school, Wuhan University, Wuhan, China, as well as 2 Department of Medical Parasitology, School of Basic Medical Science, Wuhan University, Wuhan, China. 

